# Establishment of apiary-level risk of American foulbrood through the detection of *Paenibacillus larvae* spores in pooled, extracted honey in Saskatchewan

**DOI:** 10.1038/s41598-022-12856-4

**Published:** 2022-05-25

**Authors:** Michael W. Zabrodski, Tasha Epp, Geoff Wilson, Igor Moshynskyy, Mohsen Sharafi, Lara Reitsma, Mateo Castano Ospina, Jessica E. DeBruyne, Alexandra Wentzell, Sarah C. Wood, Ivanna V. Kozii, Colby D. Klein, Jenna Thebeau, LaRhonda Sobchishin, Antonio C. Ruzzini, Elemir Simko

**Affiliations:** 1 Department of Veterinary Pathology, Western College of Veterinary Medicine, University of Saskatchewan, Saskatoon, SK Canada; 2 Department of Large Animal Clinical Sciences, Western College of Veterinary Medicine, University of Saskatchewan, Saskatoon, SK Canada; 3 Crops and Irrigation Branch, Ministry of Agriculture, Government of Saskatchewan, Prince Albert, SK Canada; 4 Department of Veterinary Microbiology, Western College of Veterinary Medicine, University of Saskatchewan, Saskatoon, SK Canada

**Keywords:** Clinical microbiology, Microbiology techniques, Entomology

## Abstract

*Paenibacillus larvae*, the causative agent of American foulbrood (AFB), produces spores that may be detectable within honey. We analyzed the spore content of pooled, extracted honey from 52 large-scale (L) and 64 small-scale (S) Saskatchewan beekeepers over a two-year period (2019–2020). Our objectives were: (i) establish reliable prognostic reference ranges for spore concentrations in extracted honey to determine future AFB risk at the apiary level; (ii) identify management practices as targets for mitigation of risk. *P. larvae* spores were detected in 753 of 1476 samples (51%). Beekeepers were stratified into low (< 2 spores/gram), moderate (2- < 100 spores/gram), and high (≥ 100 spores/gram) risk categories. Of forty-nine L beekeepers sampled in 2019, those that reported AFB in 2020 included 0/26 low, 3/18 moderate, and 3/5 high risk. Of twenty-seven L beekeepers sampled in 2020, those that reported AFB in 2021 included 0/11 low, 2/14 moderate, and 1/2 high risk. Predictive modelling included indoor overwintering of hives, purchase of used equipment, movement of honey-producing colonies between apiaries, beekeeper demographic, and antimicrobial use as risk category predictors. Saskatchewan beekeepers with fewer than 2 spores/gram in extracted honey that avoid high risk activities may be considered at low risk of AFB the following year.

## Introduction

*Paenibacillus larvae*, an endospore-forming bacterium, is the causative agent of the honey bee disease, American foulbrood (AFB)^1^. *P. larvae* endospores (hereafter referred to as spores) are infectious to newly hatched larvae^2–4^, within which they germinate, undergo massive vegetative replication, and produce hundreds of millions of new spores^5^. These spores subsequently spread throughout the colony and hive products by the caretaking actions of nurse bees^6^. Spores are exceptionally resilient, capable of maintaining infectivity for decades^7^ and able to withstand treatment by common disinfectant solutions^8,9^ and antibiotics^10^. North American apiculture is heavily reliant on the chronic and sustained use of antibiotics in the form of metaphylaxis to prevent and control outbreaks of AFB, as on-label antibiotics are generally effective at controlling the vegetative state of *P. larvae* and preventing the expression of clinical disease^11–13^. In recent years, however, several studies have documented and characterized the emergence of antibiotic resistant strains of *P. larvae* in North America^14–17^. Consequently, beekeepers may be faced with the development of clinical disease despite seemingly-appropriate and on-label antibiotic use if these resistant strains are present within their apiaries without their knowledge.

In addition to the appearance of antimicrobial resistance, the routine use of antimicrobials in apiculture is generally undesirable due to a potential persistence of residues in honey that limit marketability for human consumption, as well as the direct adverse effects they may have on larval development and survival^18,19^. It is reasonable to suggest that reducing reliance on antibiotics in North American apiculture, if done in a safe and evidence-based manner, would improve the overall sustainability and profitability of the industry as a whole. Indeed, there is an urgent need to strive toward this goal given the growing global concern regarding antimicrobial resistance in both humans and animals^20^ and the resulting, necessary regulatory changes that have directly affected beekeepers’ access to antimicrobials^21,22^.

Reducing antimicrobial reliance and ensuring more judicious use of antibiotics in apiculture requires beekeepers to have access to efficacious alternative treatments to control AFB^19^ and/or have an improved ability to confidently make evidence-based management decisions through effective risk assessment tools. If a beekeeper or beekeeping operation could incorporate reliable and logistically feasible risk assessment into their integrated pest management strategies against AFB, the recognition of a low risk of future AFB disease may signal that the use of antibiotics is temporarily unnecessary. Conversely, identification of a high risk of disease would justify the judicious use of antimicrobial metaphylaxis while simultaneously providing an opportunity to evaluate for ways to mitigate said risk.

Multiple studies have evaluated the quantification of *P. larvae* spores in bees, bottom board debris, and honey as a means of either predicting future AFB risk or detecting early clinical disease^23–31^; however, many of these studies rely on the collection of samples on an individual hive (i.e., hive-by-hive) basis. Although an individual hive approach may be useful for beekeepers managing a small number of honey bee colonies, we previously demonstrated that spores are heterogeneously distributed amongst hives within the apiaries of large-scale, commercial beekeeping operations^23,32,33^. Consequently, the ability to identify high-risk colonies using a hive-by-hive approach in these operations would require very large numbers of individually sampled hives, a practice that is logistically impossible and cost prohibitive for many large-scale, commercial beekeeping operations in North America.

It has long been known that extracted, commercial honey may be a source from which to cultivate spores of *P. larvae*^5^. Several studies have demonstrated that spore concentrations obtained from extracted, pooled (bulk) honey may be useful in AFB risk assessment; however, these studies originate from countries where the use of antimicrobials in apiculture is prohibited, test samples from beekeepers managing a relatively small number of hives, or use sampling strategies that preclude traceability to original apiaries^26,28,30,34,35^. For large-scale, commercial beekeeping operations, the collection of samples of extracted honey traceable to a specific apiary would be substantially more convenient than individual hive sampling, as sample collection could be easily integrated into the routine honey extraction workflow while spinning frames on an extractor machine^33^.

We previously showed that pooled honey collected from honey supers during routine extraction could be used as an apiary- or operation-level surrogate for more conventional, individual hive sampling strategies (i.e., adult bees or honey from brood chambers of individual colonies) for the detection of *P. larvae* spores^33^. We also demonstrated that, similar to historical studies, such samples may have prognostic value in assessing the future risk of AFB at the apiary or operation level^33^. Accordingly, we have expanded the sampling of pooled, extracted honey to a large number of both commercial and small-scale beekeepers across the province of Saskatchewan, Canada, to (i) establish prognostic reference ranges for *P. larvae* spore concentrations for the determination of AFB risk at the apiary or operation level; and (ii) use predictive modelling to identify management practices that may represent key targets for intervention and mitigation of AFB risk. The results of this study will enable enhanced risk assessment for future AFB at the apiary or operation level, along with potential intervention strategies to mitigate AFB risk; accordingly, beekeepers and beekeeping operations in North America will be better equipped to safely reduce their reliance on antimicrobials in the prevention and control of AFB. This evidence-based and logistically feasible approach will help to ensure not only the continued sustainability of the industry, but will also serve to reduce antimicrobial resistance within North American apiculture.

## Materials and methods

### Sample collection and questionnaires

To ensure province-wide representation across Saskatchewan, beekeepers approached for study enrollment belonged to one of five subjectively bordered regions: Saskatoon region (defined by a 250 km^2^ area around the municipality of Saskatoon), Regina region (defined by a 250 km^2^ area around the municipality of Regina), Northeast (longitudinally defined from Highway 2 and the Eastern provincial border, latitudinally defined from Highway 5 to the town of La Ronge—approximately 1.06 × 10^5^ km^2^), Northwest (longitudinally defined from the Western provincial border to Highway 2, latitudinally defined from Highway 14 to the town of Beauval—approximately 9.68 × 10^4^ km^2^), and South (south of Highways 14 and 5, excluding the area defining the Regina region—approximately 2.04 × 10^5^ km^2^). Enrolled beekeepers and beekeeping operations were stratified into two demographic categories: small-scale beekeepers, defined as those with fewer than 100 hives (hereafter referred to as S beekeepers; range 1 to 98, average = 16, standard deviation = 24, median = 8), and large-scale beekeepers and beekeeping operations, defined as those managing greater than or equal to 100 hives (hereafter referred to as L beekeepers; range 100 to 6300, average = 1578, standard deviation = 1584, median = 935). Demographic variables used to describe these two categories included total number of hives, number of apiaries, full-time/part-time beekeeping status, percentage of income derived from beekeeping, and employment of staff. S beekeepers typically refer to those managing honey bees part-time or as a special interest (i.e., hobby), whereas L beekeepers tend to refer to commercial beekeeping operations performing beekeeping on a large scale and employing staff.

Sample collection spanned the honey-producing seasons of 2019 and 2020. All S beekeepers were solicited for honey samples from each of the sampling years, whereas L beekeepers were solicited once in 2019, and a selected subset solicited again in 2020. This selected subset included beekeepers with 2019 maximum spore concentrations greater than or equal to five spores per gram of honey, beekeepers that had experienced at least one case of AFB within the previous 10 years (determined based on questionnaire—see below), select beekeepers (n = 3) with low 2019 spore concentrations (i.e., less than five spores per gram of honey) not currently using antimicrobial metaphylaxis, select beekeepers (n = 3) with low 2019 spore concentrations currently using antimicrobial metaphylaxis, and additional select beekeepers (n = 2) with low 2019 spore concentrations managing very large commercial beekeeping operations (approximately 5000 hives). The Canadian Council on Animal Care (CCAC) does not require permission for research on insects; however, all sampling procedures were performed in accordance with the Saskatchewan Apiaries Act.

Samples were collected from the last honey harvest (i.e., late August to early September) to ensure as wide a gap in time as possible from spring treatment with antibiotics. S beekeepers were asked to submit a single sample of extracted honey per year that was collected from their largest apiary. L beekeepers were asked to submit a total of 18 extracted honey samples per year using an adaptation of a two-stage sampling protocol^36^. Specifically, L beekeepers randomly selected six geographically separate apiaries or lots and collected three honey samples from each. For any given apiary or lot, each sample was collected from a separate extractor load to avoid repeated sampling of frames and hives. With this strategy, we estimated that these three unique honey samples represent between nine and 18 hives within an apiary or lot^33^. Here, the use of the term ‘apiary’ is synonymous with the North American term ‘bee yard’, whereas a lot refers to geographically clustered apiaries that are extracted together as a single unit^33^. Upon receipt, all samples were stored at room temperature until quantitative bacterial cultures of *P. larvae* spores were performed.

In addition to the submission of honey samples, beekeepers were asked to complete an accompanying questionnaire regarding their beekeeping experience, current management practices, and history with AFB^30^ (Fig. S1). Questionnaires were distributed in May of 2020, the majority of which were completed throughout the remainder of the year. Accordingly, collected data reflected routine beekeeper practices during 2019 and 2020. Collection and storage of personal information within these questionnaires was done in accordance with the requirements set by the Behavioural Research Ethics Board (Beh-REB) of the University of Saskatchewan (Ethics Approval ID 1868), and required the signed consent of participants. In 2020, participating S and L beekeepers that developed clinical signs consistent with AFB were identified by one or more of the following: direct verbal communication, reporting to and/or inspection by the provincial specialist in apiculture, and/or recording of occurrence of AFB in the study questionnaire. In 2021, L beekeepers that developed clinical signs consistent with AFB were identified as those that had reported to and/or been inspected by the provincial specialist in apiculture. Determination of the 2021 AFB status of S beekeepers was not attempted. L beekeepers with subjectively large discrepancies in spore concentrations between 2019 and 2020 were contacted in an attempt to identify any changes in management practices (including antibiotic use), overall colony strength, and/or prevalence of other diseases that could potentially explain these differences.

### Media preparation and *P. larvae* cultivation

A complex MYPGP medium was used for the cultivation of *P. larvae* spores, combining conventional MYPGP medium^37,38^ with germination agonists (i.e., L-tyrosine and uric acid) reported by Alvarado et al. (2013)^39^. For a complete description of medium preparation, hereafter referred to as enhanced MYPGP medium, please refer to our previous study^33^. Poured plates were stored at 4 °C until use. Any unused plates older than 2 months of age were discarded.

To cultivate *P. larvae* spores from honey, samples were processed as previously described^33^. Briefly, approximately 20 g of honey was weighed out in a falcon tube and mixed with 20 mL of sterile water. The exact weight of honey was recorded and used for the subsequent calculations. This mixture was shaken overnight at 37 °C to allow for honey dissolution, and the resulting suspension was centrifuged at 6000 g for 40 min at 21°C^1,37^. Supernatant was discarded, the pellet re-suspended in 2 mL of sterile water, and the final suspension vortexed for 20 s. A portion of this suspension was heat-treated at 85 °C for 15 min and cooled to room temperature^40^. Heat-treated suspension was spread across three technical replicate plates of enhanced MYPGP medium (200 μL per plate, 600 μL total), and 200 μL of unheated suspension was spread onto a fourth plate as a control^33^. Plates were incubated at 37 °C with 5% CO_2_ for seven days^1,37^. For unheated control plates, bacterial colonies morphologically consistent with *P. larvae* were counted after 72 h of incubation before overgrowth by contaminant bacteria and fungi could occur, then counted again after seven days on plates that were not overgrown by contaminant microorganisms. Bacterial colonies consistent with *P. larvae* were counted on all plates after seven days of incubation, and the number of colonies were averaged across the three technical replicates. Spore concentrations were calculated under the assumption that each bacterial colony was representative of a single *P. larvae* spore^1,24^. If one or more replicate plates of heat-treated suspension for a given sample cultivated greater than 100 colonies of *P. larvae*, the suspension was re-plated and incubated following serial, ten-fold dilutions^41^. Any bacterial colonies with an equivocal *P. larvae* morphology were sub-cultured to thoroughly assess morphological characteristics. If colony identification remained ambiguous following sub-culture, suspect colonies were identified using matrix-assisted laser desorption/ionization-time of flight mass spectrometry (MALDI-TOF MS) by Prairie Diagnostic Services Inc., Western College of Veterinary Medicine, Saskatoon, Saskatchewan.

### Establishment of prognostic reference ranges for honey

To assess the ability of pooled, extracted honey to predict the risk of an outbreak of AFB at the apiary or operation level, spore concentrations were stratified into low, moderate, and high categories using natural breakpoints (thresholds) within the combined dataset of S and L beekeeper samples. Tentative categories for future AFB risk (i.e., low, moderate, and high risk) corresponded to low, moderate, and high spore concentrations. Beekeepers were assigned to a risk category for a given year based on their highest (maximum) spore concentration honey sample from that year. Final thresholds for risk categories were determined using data on future occurrences of AFB, the historical occurrence of AFB for each beekeeper, and prediction graphs generated from the final predictive model to evaluate its ability to differentiate between the different risk categories.

### Identification of risk factors and predictive modelling

Questionnaire responses were transcribed and coded using Microsoft Excel and analyzed using STATA software (Version 15.1; Statacorp. LLC). Coding differentiated between responses that were inapplicable for a particular respondent (based on their demographic or previous questionnaire answers), and those responses that were truly missing (not answered when they should have been). For ambiguous or incomplete responses, clarification was obtained directly from the responding beekeeper whenever possible. If beekeepers were unavailable for follow-up, ambiguous or incomplete responses were encoded with the lowest or most conservative value possible to ensure as conservative an interpretation as possible for analysis. For responses provided as a range of numerical values, the average of these values was used for analysis.

To identify management practices that may be important in predicting a beekeeper’s risk of AFB disease, individual management variables were analyzed using ordered logistic regression comparing both combined moderate and high risk (i.e., moderate/high) to low risk, and high risk to combined moderate and low risk (i.e., moderate/low). A single odds ratio was reported for each individual variable following verification of proportionality using the Wolfe Gould proportional odds test. Variables with odds ratios with greater than 65 observations and a p-value of less than 0.3 were used as candidate explanatory variables to generate a predictive model using a beekeeper’s highest category of risk of AFB across 2019 and 2020 as an outcome. Antibiotic use was included as a model variable regardless of its univariable p-value due to its possible role as an important confounder. Models were differentiated from one another by pseudo R-squared values, number of observations, and overall model stability. Within each proposed model, attainment of the highest pseudo R-squared value possible was prioritized over an individual variable’s retainment of statistical significance once added. Explanatory variables within the final model (i.e., highest pseudo R-squared model with model stability) were considered to be management factors relevant for the prediction of risk for future AFB disease.

In order for beekeepers to utilize identified risk factors as points of intervention to mitigate AFB risk, management practices were categorized into one of the following: prevention of AFB, control of AFB, and general (non-specific). Variables under prevention of AFB included frequency of inspection of brood frames, number of brood frames evaluated during inspection, investigation of winter dead-outs, training of staff in disease recognition and diagnosis, current antimicrobial metaphylaxis use, and questions relating to the handling and movement of brood frames, hives, and extracted honey supers within and between apiaries. Variables under control of AFB included destruction of suspect and/or confirmed AFB colonies and hives, reactionary use of antimicrobials following identification of suspect and/or confirmed AFB colonies and hives, use of a quarantine or isolation apiary, and reporting of suspect cases of AFB to the provincial specialist in apiculture. General management practices included mobility of hives and apiaries (i.e., for pollination services), indoor overwintering, barrel feeding, and the purchase/selling of used equipment and colonies.

## Results

### Summarization of honey samples over study period

A total of 116 Saskatchewan beekeepers were represented in at least one of the honey-producing seasons within the study period (2019 and/or 2020), consisting of 64 S and 52 L beekeepers. Sixty-one S beekeepers were represented in 2019, and 45 were represented in 2020. Forty-three S beekeepers provided samples from both sampling years, 18 provided samples from 2019 only, and the remaining three S beekeepers provided a sample from 2020 only. Fifty-two L beekeepers were sampled in 2019, and a subset of these (27 L beekeepers) were sampled again in 2020. Together, these 116 S and L beekeepers owned approximately 75% (over 82,000 out of ~ 110,000) of Saskatchewan’s registered honey bee hives during the study period, consisting of over 56,000 honey-producing hives, approximately 21,000 nucleus hives, and 5000 hives of unspecified designation^42^.

Over the study period of 2019 and 2020, a total of 1476 honey samples were tested for spores of *P. larvae*, consisting of 111 honey samples submitted by S beekeepers and 1365 samples submitted by L beekeepers. Sixty-five S beekeeper samples from 2019 and 46 samples from 2020 were tested. For L beekeepers, 888 samples were tested from 2019 and 477 samples were tested from 2020.

### Establishment of prognostic thresholds for *P. larvae* spore concentrations in honey

**Figure 1:**
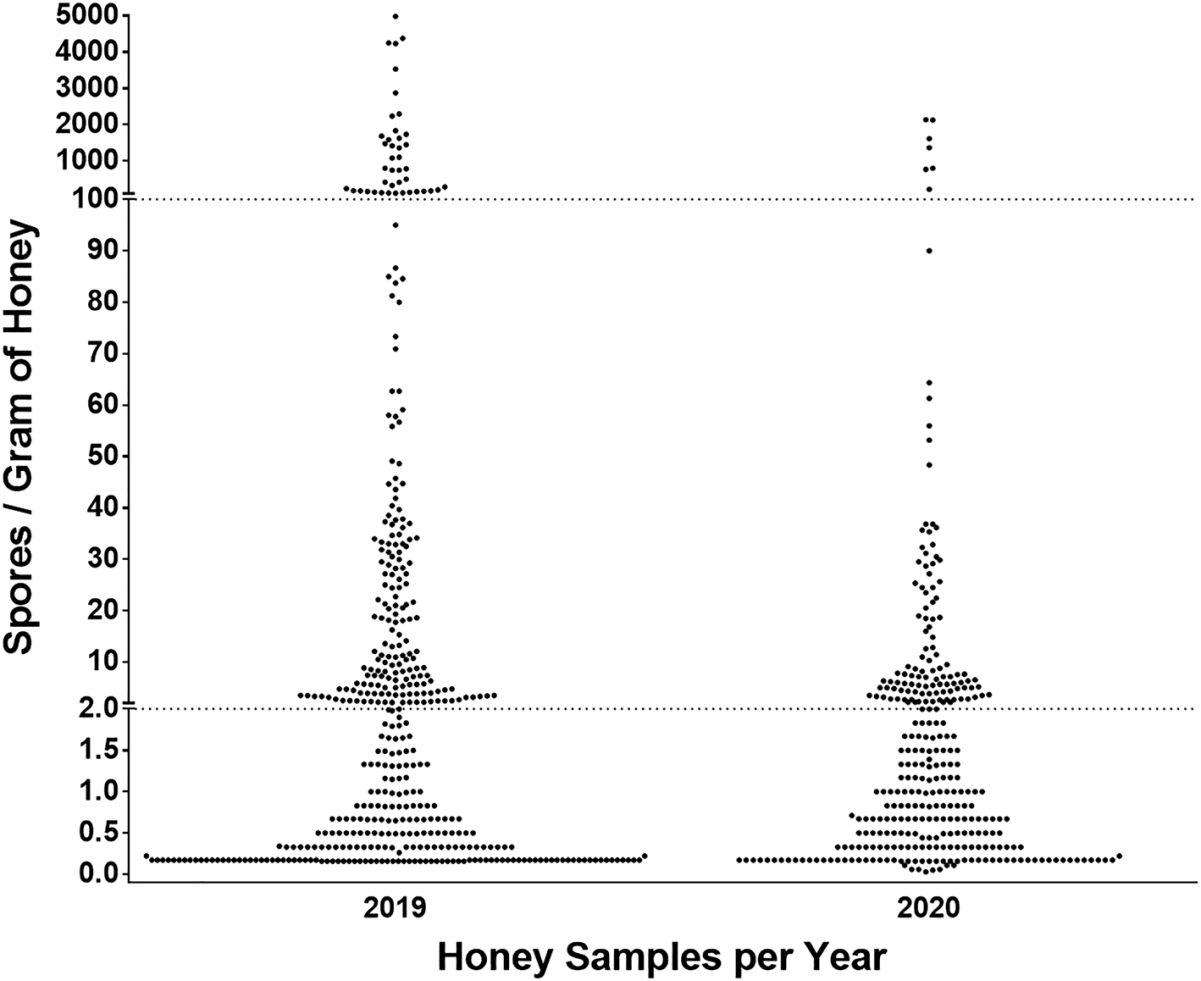
Samples of pooled, extracted honey with detectable spores of *Paenibacillus larvae*. Plotted values represent 753 honey samples (out of 1476) collected from both small-scale (S) and large-scale (L) beekeepers across two honey-producing seasons (2019–2020) that had detectable spores of *P. larvae* (range = 0.1–4983 spores per gram of honey). Samples with no detectable spores are not plotted. Dotted lines represent intuitive visual breakpoints (thresholds) with cut-off values delimiting categories of spore contamination where two spores per gram of honey differentiates low from moderate concentrations and 100 spores per gram of honey differentiates moderate from high concentrations.

Spores of *Paenibacillus larvae* were detected in 753 of 1476 honey samples (51%) across the entire study period. Of those samples with detectable spores, 718 were from L beekeepers (52.6% of all L beekeeper samples) and 35 were from S beekeepers (31.5% of all S beekeeper samples). The visual distribution of spore concentrations of all samples with detectable spores is presented in Fig. 1. The visual threshold delimiting low spore concentrations from moderate spore concentrations was established intuitively at two spores per gram of honey, and the threshold delimiting moderate from high was established intuitively at 100 spores per gram of honey. Across 2019 and 2020, 28 of 35 (80.0%) of S beekeeper samples with detectable spores had < 2 spores per gram of honey, 6 of 35 samples (17.1%) had 2 −  < 100 spores per gram of honey, and 1 of 35 (2.9%) had a concentration of spores ≥ 100 spores per gram of honey (maximum of 156 spores per gram). Across 2019 and 2020, 396 of 718 (55.2%) of L beekeeper samples with detectable spores had < 2 spores per gram of honey, 274 of 718 samples (38.2%) had 2 −  < 100 spores per gram of honey, and 48 of 718 samples (6.7%) had ≥ 100 spores per gram of honey (maximum of 4983 spores per gram).

**Figure 2:**
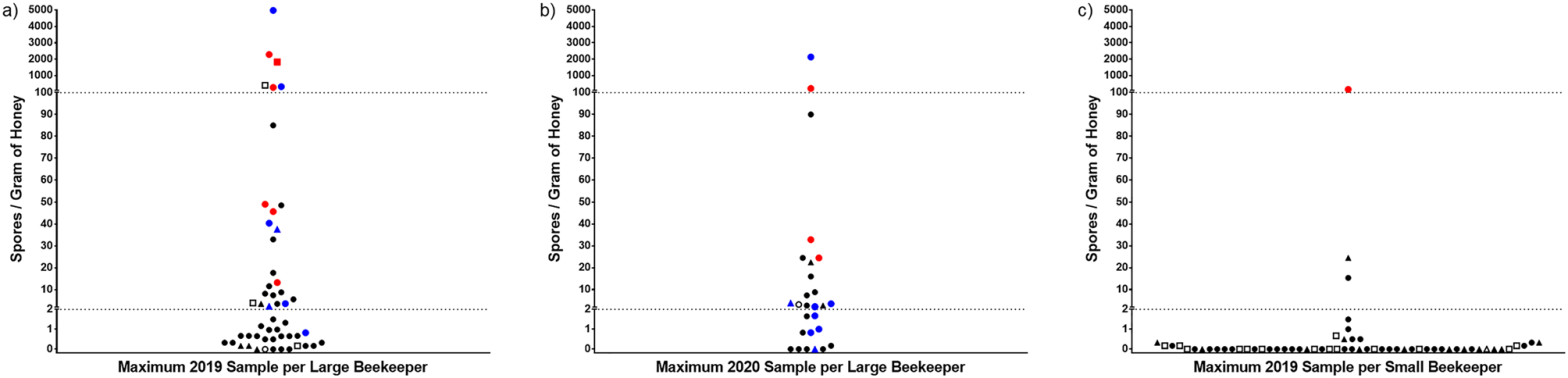
Maximum spore concentrations of *Paenibacillus larvae* in pooled, extracted honey relative to future and historical AFB occurrence. (**a**) Maximum 2019 spore concentrations of large-scale (L) beekeepers; (**b**) maximum 2020 spore concentrations of L beekeepers; (**c**) maximum 2019 spore concentrations of small-scale (S) beekeepers. Samples highlighted in red indicate occurrence of AFB in the subsequent year from the year the sample was collected in. Samples highlighted in blue indicate historical occurrence of AFB within the preceding four years. Samples with open centers are those missing information on future and/or historical AFB occurrence. Dotted lines represent cut-off values delimiting categories of future AFB risk where two spores per gram of honey differentiates low from moderate risk and 100 spores per gram of honey differentiates moderate from high risk. Circles represent current antibiotic use or cessation of antibiotic use after 2017. Triangles represent those beekeepers who have never used antibiotics or cessation during or prior to 2017. Squares represent missing information on antibiotic use.

Potential categories of future AFB risk were chosen using the intuitive thresholds of two and 100 spores per gram of honey delimiting low, moderate, and high spore concentrations. Beekeepers were assigned to low, moderate, and high categories of risk each year based on their single, highest spore concentration in that given year (Fig. 2). In 2019, 27 of 52 L beekeepers (51.9%) were assigned to low risk of AFB, 19 of 52 (36.5%) were assigned to moderate risk, and six of 52 (11.5%) were assigned to high risk of AFB. For the selected subset of L beekeepers in 2020, 11 of 27 (40.7%) were assigned to low risk, 14 of 27 (51.8%) were assigned to moderate risk, and two of 27 (7.4%) were assigned to high risk of AFB. For S beekeepers in 2019, 58 of 61 (95.1%) were assigned to low risk of AFB, two of 61 (3.3%) were assigned to moderate risk, and one S beekeeper was assigned to high risk of disease. In 2020, 41 of 45 (91.1%) of S beekeepers had a low risk of AFB, while the remaining 4 of 45 (8.9%) were assigned moderate risk.

To determine if categories of low, moderate, and high spore concentrations corresponded with low, moderate, and high risk of AFB disease, respectively, L beekeepers received follow-up throughout 2020 and 2021 and S beekeepers received follow-up in 2020 to identify occurrences of AFB. Six out of 49 L beekeepers sampled in 2019 (that provided follow-up) reported AFB occurrence in 2020; out of these six, zero of 26 (0%) were assigned to low risk, three of 18 (17%) to moderate risk, and three of five (60%) to high risk (Fig. 2a). Out of the 27 L beekeepers sampled in 2020, three reported occurrences of AFB directly to the provincial specialist in apiculture in 2021. Out of these three, zero of 11 (0%) were assigned to low risk, two of 14 (14.2%) to moderate risk, and one of two (50%) to high risk (Fig. 2b). The three L beekeepers that reported AFB in 2021 also reported AFB in 2020. One out of 52 S beekeepers sampled in 2019 (that provided follow-up) reported AFB occurrence in 2020; this S beekeeper had 156 spores per gram of honey and was the only S beekeeper assigned to high risk in 2019 (Fig. 2c). Overall, the seven occurrences of AFB identified in 2020 and three occurrences of AFB identified in 2021 included all official cases of AFB across the entirety of Saskatchewan that were reported to the provincial specialist in apiculture in each of these years.

### Relation of *P. larvae* spore concentrations to historical occurrence of American foulbrood

Through available questionnaire data, spore concentrations in pooled, extracted honey were compared to any historical occurrence of AFB during the preceding four years of the date of sample collection (Fig. 2). These four preceding years included the sampling year itself, as samples were collected at the very end of the honey-producing season and would reflect changes earlier in the year. Nine out of 45 L beekeepers sampled in 2019 (that provided historical information) reported AFB occurrence from 2016 to 2019 (inclusive); out of these nine, one of 25 (4%) was assigned to low risk, five of 16 (31.3%) to moderate risk, and three of four (75%) to high risk (Fig. 2a). Eleven out of 26 L beekeepers sampled in 2020 (that provided historical information) reported AFB occurrence from 2017 to 2020 (inclusive); out of these 11, four of 11 (36.4%) were assigned to low risk, five of 14 (35.7%) to moderate risk, and two of two (100%) to high risk (Fig. 2b). None of 48 S beekeepers sampled in 2019 that provided information regarding historical AFB, including the single S beekeeper with AFB in 2020, had ever previously detected a case of AFB within their hives.

### Comparison of 2019 and 2020 *P. larvae* spore concentrations in individual L beekeepers

**Figure 3:**
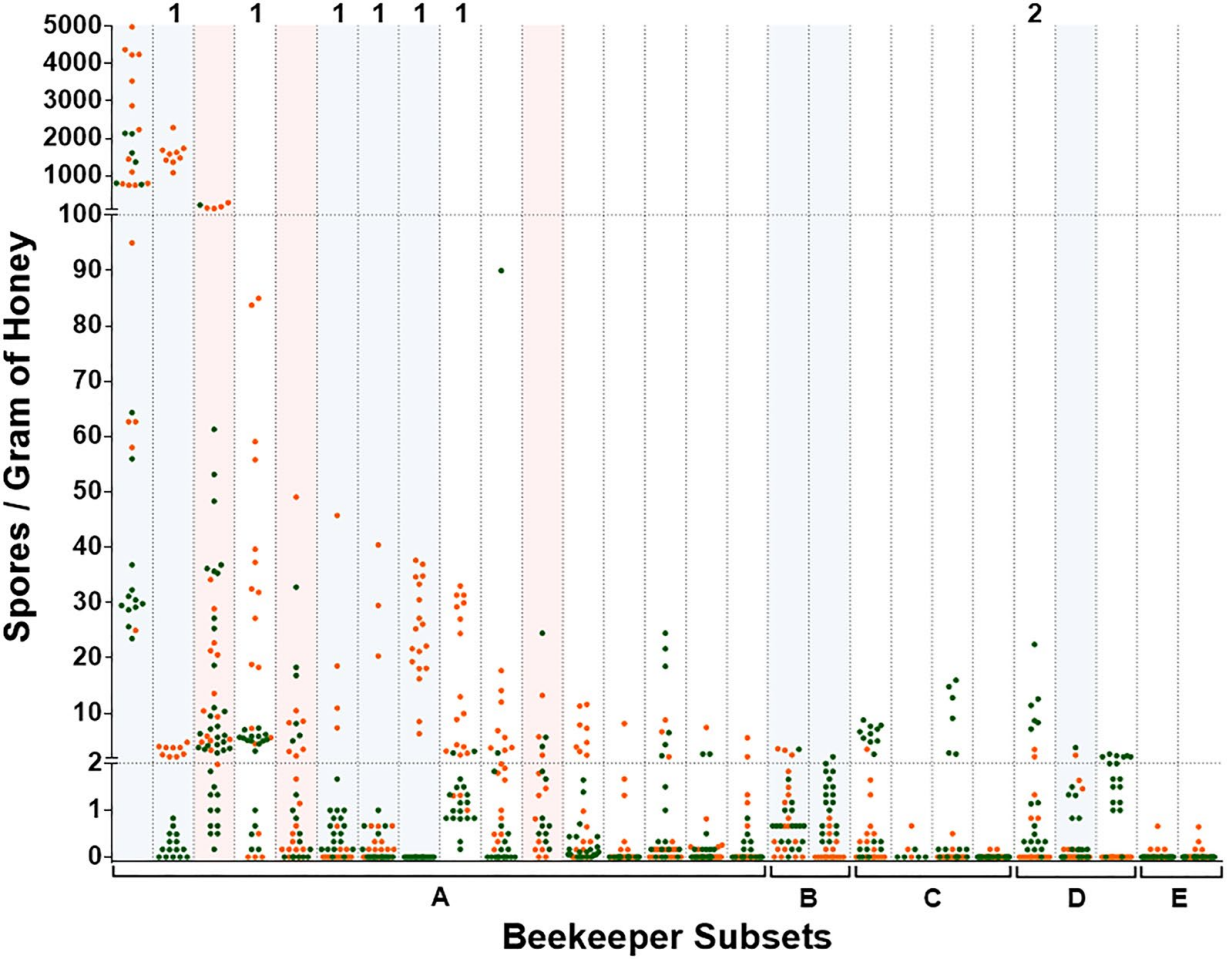
*Paenibacillus larvae* spore concentrations in extracted honey of large-scale (L) beekeepers sampled in both 2019 and 2020. Orange dots indicate spore concentrations in honey samples collected in 2019 and green dots for 2020. L beekeepers are separated from one another by vertical dashed lines. Beekeepers highlighted in light red are those that reported AFB in 2021. Beekeepers highlighted in light blue are those that reported a historical occurrence of AFB within the previous four years (2017–2020). L beekeepers were selected for repeat (2020) sampling by meeting one of the following subset criteria: A = maximum 2019 spore concentration greater than or equal to five spores per gram of honey; B = at least one case of AFB within the previous 10 years; C = select beekeepers with a maximum 2019 spore concentration less than five spores per gram of honey currently using antibiotics as a part of AFB management; D = select beekeepers with a maximum 2019 spore concentration less than five spores per gram of honey not currently using antibiotics; E = select beekeepers with a maximum 2019 spore concentration less than five spores per gram managing very large commercial beekeeping operations (approximately 5000 hives). 1 = L beekeepers that provided follow-up regarding decrease in spore concentrations between sampling years. 2 = L beekeeper with apiaries within flying distance of reported case of AFB between 2019 and 2020 sampling years.

The distributions of spore concentrations for the selected subset of L beekeepers that were sampled in both 2019 and 2020 are presented in Fig. 3. Seventeen of 27 (63.0%) L beekeepers did not change risk categories between 2019 and 2020 based on the maximum spore concentrations of *P. larvae* detected in pooled, extracted honey. Seven of 27 (25.9%) were in a lower risk category in 2020 relative to 2019. Of these, six of seven decreased from moderate risk to low risk, and one L beekeeper decreased from high risk to low risk. Three of 27 (11.1%) were in a higher risk category in 2020 relative to 2019, all of which increased from low risk to moderate risk.

Six L beekeepers with relatively large decreases in maximum spore concentrations were contacted for follow-up. One or more apiaries were sampled during both years for four of six of these L beekeepers. Three of six L beekeepers implemented one or more of the following management changes in an active and concerted attempt to reduce their degree of spore contamination: discarding most to all of brood comb and equipment from colonies that died overwinter (winter dead-outs), performing more thorough inspections of winter dead-outs, replacing as much old brood comb as possible across the entirety of the beekeeping operation, increasing the frequency and thoroughness of brood chamber inspections in live colonies, implementing operation-wide use of antimicrobial metaphylaxis, and/or improving on-label adherence of antimicrobial use. The remaining three L beekeepers did not actively implement any changes specific to AFB management, although one of these three reported an increased use of queens with improved hygienic behavior between 2019 and 2020.

The apiaries of one of the three L beekeepers whose spore concentrations increased between 2019 and 2020 were found to be within close proximity to a confirmed case of AFB that occurred between these sampling events. This case of AFB belonged to a beekeeper not included in this study. Clinical disease was confirmed by the provincial specialist in apiculture.

### Identification of risk factors and predictive modelling

Evaluation of the relationship between individual management practices and the three risk categories was performed using ordered logistic regression, where combined moderate and high (moderate/high) risk was compared to low risk, and high risk was compared to combined low and moderate (low/moderate) risk. These outcome risk categories were based on a beekeeper’s highest category of risk of AFB across both 2019 and 2020. Odds ratios for individual management practices are summarized in Table 1. For L beekeepers, the odds of being at or in a higher risk category(ies) than being in a lower risk category(ies) (i.e., higher risk categorization) is 9.3 times that of S beekeepers (N = 97, 95% confidence interval [CI] = 3.1–27.3, p < 0.001). Additionally, for full-time beekeepers, the odds of being at or in a higher risk category(ies) than being in a lower risk category(ies) (i.e., higher risk categorization) is 8.4 times that of part-time beekeepers (N = 96, 95% CI = 3.0–23.4, p < 0.001). Due to the disparity between S and L beekeepers, we chose to use beekeeper size in all models to account for these differences as a possible confounder.

**Table 1:** Odds ratios for individual management practices with moderate/high and high future AFB risk categories as an outcome. Only variables with > 65 observations and a p-value of < 0.3 are included, with the exception of Current/Recent Antibiotic Use. For each variable, the categories analyzed are described with the reference category displayed in brackets.

Management Variable	Categories (Reference in Brackets)	Observations (N)	Odds Ratio (OR)*	95% Confidence Interval	p-value
Occurrence of AFB Within Previous Five Years	Yes (no)	93	11.5	3.3, 40.6	< 0.001
Beekeeper Size Code	Large-scale (Small-scale)	97	9.3	3.1, 27.3	< 0.001
Inspection Personnel	Both Owner and Staff (Either Owner or Staff)	96	8.6	3.3, 22.9	< 0.001
Full-time/ Part-time Status	Full-time (Part-time)	96	8.4	3.0, 23.4	< 0.001
Highest Confidence Score with AFB Recognition between Owner and Staff	Very Confident (Somewhat or Not Confident)	97	7.5	2.7, 20.8	< 0.001
Confidence of Owner with AFB Recognition	Very Confident (Somewhat or Not Confident)	97	6.2	2.3, 16.5	< 0.001
Percentage of Hives Overwintered Indoors	Continuous—each increment equal to 10%	94	1.2	1.1, 1.4	0.001
Movement of Colonies between Apiaries	Sometimes or Often (No)	97	4.3	1.7, 11.1	0.002
Handling of Extracted Honey Supers	No Regard (Try to Retrun Supers to the Same Apiary or Colony)	95	3.4	1.2, 9.3	0.02
Historical Purchase of Used Equipment	Yes (No)	92	8.6	1.1, 68.2	0.04
Selling of Equipment to Other Beekeepers	Yes (No)	96	2.6	0.99, 6.6	0.05
Mobility of Hives	Mobile (Stationary)	97	0.45	0.16, 1.2	0.11
Current/Recent Antimicrobial Use	Current Use/Stopped Use After 2017 (Never used/Stopped Use in 2017 or Before)	96	1.6	0.55, 5.02	0.36

*All odds ratios meet the assumption of proportional hazards.

Following univariate analysis, candidates for explanatory variables in a predictive model included beekeeper size, current/recent antimicrobial use, percentage of hives overwintered indoors, movement of brood frames between apiaries, movement of honey-producing colonies between apiaries, mixing of extracted honey supers between apiaries, confidence in recognition of AFB clinical signs, historical purchase of used equipment, and selling of equipment to other beekeepers. Despite a univariate p-value of 0.36, the variable of current/recent antimicrobial use was included in all models due to its potential role as a confounder. Proposed predictive models using a combination of these variables are detailed in Table 2. Of the two models, model 1 was selected as the final predictive model due to a higher pseudo R-squared value relative to model 2. This final model (model 1) included a single demographic variable (beekeeper size), three management practices related to the prevention of AFB (current/recent antibiotic use, confidence in AFB recognition, and movement of colonies between apiaries), and two general management variables (overwintering of hives indoors and the historical purchase of used equipment). The ability of the final model (model 1) to predict a beekeeper’s risk category for AFB was evaluated by comparing the model’s predictions to each beekeeper’s designated, maximum risk category over the two-year sampling period (2019 and 2020). Model 1 correctly predicted the low risk category (i.e., < 2 spores/gram of honey) for 56 of 61 beekeepers (92%) assigned to this category by maximum spore concentration. This model correctly predicted the moderate risk category for 11 of 22 beekeepers (50%), and correctly predicted the high risk category for zero of four beekeepers (0%). When moderate and high risk beekeepers were combined into a single grouping (i.e., moderate/high risk), the predictive ability of the model improved to 14 of 26 beekeepers (54%). Due to the model’s ability to differentiate between low risk and higher risk categories, the lower prognostic threshold was maintained at two spores per gram of honey. There was insufficient differentiation between moderate and high risk to reliably alter the upper prognostic threshold of 100 spores per gram of honey.

**Table 2:** Proposed ordered logistic regression models for prediction of a beekeeper’s risk of AFB. Combined medium and high risk categories were compared to the low risk category (less than two spores per gram of honey), and the high risk category (greater than or equal to 100 spores per gram of honey) was compared to combined low and medium risk. Models were differentiated from one another by pseudo R-squared values, number of observations, and overall model stability.

Proposed model	Variables included	Observations (N)	Pseudo R-squared	Model stability
1	Beekeeper Size Code Current/Recent Antimicrobial Use Percentage of Hives Overwintered Indoors Purchase of Used Equipment Movement of Colonies between Apiaries Highest Confidence Score for AFB Recognition Recent Occurrence of AFB	87	0.2580	Stable
2	Beekeeper Size Code Current/Recent Antimicrobial Use Percentage of Hives Overwintered Indoors Movement of Colonies between Apiaries Highest Confidence Score for AFB Recognition Recent Occurrence of AFB	91	0.2351	Stable

## Discussion

In this study, we have provided evidence that *P. larvae* spore concentrations in pooled, extracted honey may predict a beekeeper’s risk of clinical AFB disease in the following year. By establishing prognostic thresholds at two spores per gram of honey and 100 spores per gram of honey, we have identified three demonstrably different categories of low, moderate, and high risk for future AFB at the apiary or operation level. In addition, we created a predictive model that identified several management practices that may represent key targets for intervention and mitigation of AFB risk within the context of antibiotic-reliant apiculture. Through an understanding of both *P. larvae* spore concentrations within their apiaries and management practices that may be contributing to the risk of AFB, beekeepers will be better equipped to make evidence-based decisions to prevent and control AFB, and will improve their ability to use antimicrobials in a more judicious manner.

The use of pooled, extracted honey to correlate spore concentrations of *P. larvae* with the occurrence of AFB has been documented as early as 1978, when Hansen and Rasmussen (1986) requested beekeepers to submit samples of honey from their annual harvest^34^. In this study, the authors used the number of *P. larvae* colonies cultivated on J-agar plates following heat treatment and direct inoculation to generate categories of spore concentrations ranging from no detectable spores to over 600000 spores per 5 g of honey. There were very few occurrences of AFB the following year in those apiaries with no detectable spores (two out of 476 apiaries), whereas the highest proportion of apiaries that went on to develop AFB the next year were in the highest spore concentration category (five of 21 apiaries)^34^. In our present study, the range of spore concentrations was much smaller than Hansen and Rasmussen (1986), although this could potentially reflect differences in methodology of sample collection, culture medium, antibiotic use amongst the sampled beekeepers (no antibiotic use was mentioned by Hansen and Rasmussen), and/or the amount of honey used for cultivation (approximately 20 g in this study, 0.08 g in Hansen and Rasmussen’s). Regardless, our results corroborate the use of pooled, extracted honey as a tool to determine future AFB risk. In addition, we established useful prognostic thresholds to guide evidence-based decisions regarding AFB management. In comparison to Hansen and Rasmussen’s study, where spores were detected in 11% of samples and two of 11 cases of AFB were from apiaries with no detectable spores, we detected spores in 51% of samples and all reports of AFB were from beekeepers with detectable spores, which may reflect a higher sensitivity for spore detection with our methodology.

In addition to future risk, spore concentrations in pooled, extracted honey may also reflect the overall severity and chronicity of AFB within a beekeeping operation. Hornitzky and Clark (1991) used bulk honey samples to cultivate *P. larvae* colonies on sheep blood agar following heat treatment and centrifugation^35^. Colony counts on plates were scored into 1 + , 2 + , and 3 + categories corresponding to severity of contamination, where 1 + corresponded to 1 to 20 colonies on a plate, 2 + to 21 to 50 colonies, and 3 + to over 50 colonies. The authors found that 56.4% of honey samples assigned a 1 + score, 78.6% of 2 + honey samples, and 100% of 3 + honey samples were from beekeepers with either active or historical AFB disease^35^. Although it was not possible to correlate *P. larvae* colony counts on culture plates to actual spore concentrations in honey samples in Hornitzky’s study, in our present study, we also found that 100% of beekeepers assigned to our highest risk category (i.e., those with a maximum spore concentration greater than or equal to 100 spores per gram of honey) had reported either the subsequent development of AFB or a recent historical occurrence of the disease. These findings suggest that beekeepers assigned a high risk of future AFB are also likely to be dealing with chronic contamination within their hives or operations.

The findings of this current study will be relevant to beekeepers across western Canada, not just those within the borders of Saskatchewan. Pernal and Melathopoulos (2006) previously analyzed *P. larvae* spore content in pooled, extracted honey from commercial beekeeping operations in the province of Manitoba, Canada^26^. Located immediately adjacent to the province of Saskatchewan, management practices of beekeepers in Manitoba are considered comparable to those of the beekeepers in this study. In fact, spore concentrations detected in honey by Pernal and Melathopoulos were within the same range of magnitude as in our study. The authors evaluated the use of adult bees from brood chambers and honey collected from settling tanks as an indicator of the severity of active disease within a beekeeping operation. Although they successfully demonstrated that the concentration of spores in honey samples was positively correlated with the number of colonies exhibiting clinical signs of AFB, they ultimately found that bees were a stronger predictor of current, active disease status^26^. Pernal and Melathopoulos (2006) reasoned that pooled honey may be more valuable as a tool to identify operations at high risk of developing AFB, suggesting that threshold levels may be able to screen out operations with no apparent risk. In our study, we have established these prognostic thresholds for honey samples coming from hives managed very similarly to those in Manitoba, although there are important differences between our work and that of Pernal and Melathopoulos (2006). We analyzed unique samples of pooled, extracted honey collected directly from extractor loads in a two-stage sampling strategy^36^, as opposed to collecting samples in 3000 kg increments from settling tanks^26^. We also used an enhanced MYPGP culture medium instead of modified PLA medium^26^. Nevertheless, we believe that our prognostic thresholds from pooled, extracted honey are applicable to beekeepers across western Canada, given the uniformity of management practices across the country.

Many strategies and interventions to prevent and control AFB are universally accepted as standard of practice, regardless of whether or not the region in question uses antimicrobials in beekeeping^28,43–45^. These strategies include frequent and thorough inspection of brood chambers, evaluation of deceased colonies, annual replacement of brood comb, training of staff in disease recognition and diagnosis to improve early detection, and the practice of biosecurity and quarantine in the handling and movement of equipment within and between apiaries. Several of the L beekeepers whose spore concentrations dramatically decreased between 2019 and 2020 reported implementation of one or more of these strategies upon learning of high spore concentrations within their operations in 2019. In particular, these L beekeepers emphasized the replacement of large amounts of old brood comb, an increased frequency and thoroughness of inspection of winter dead-outs and/or live colonies, and implementation or improved adherence to on-label antimicrobial metaphylaxis as major changes made in management. Further investigation of the potential ability of these mitigative interventions to rapidly decrease spore concentrations in antibiotic-reliant apiculture are warranted, as they may represent key intervention strategies for those beekeepers assigned a high risk of AFB. Recent work by Locke et al. (2019) is promising in this regard. They demonstrated that the consistent quarantine of individual colonies with high levels of spores, a process involving the replacement of all old hive materials including comb, successfully reduced the percentage of apiaries with detectable *P. larvae* spores from 74 to 4% over a period of 5 years within a modestly-sized beekeeping operation (between 560 and 670 hives amongst 56 apiaries) in Sweden^23^.

In addition to the above mentioned strategies, our predictive modelling supports the value of biosecurity between the apiaries of different beekeepers, as well as between the apiaries of a single beekeeper, as we identified the historical purchase of used equipment and the movement of honey-producing colonies between apiaries, respectively, as management practices correlated with a moderate to high risk category (Table 3). Beekeepers should therefore make efforts to minimize both the purchase of used equipment and the movement of equipment and colonies between apiaries whenever possible^44,45^. If used equipment must be purchased, then professional inspection by a specialist in apiculture should be performed prior to purchase. Newly introduced, used equipment should also be established in separate (quarantine) apiaries in order to minimize any risk of spore transmission to other apiaries or hives. At the very least, all introduced equipment and hives with colonies should be appropriately labeled or otherwise identified to improve traceability in the event of an outbreak.

**Table 3:** Proposed significance of variables in final model of prediction of future AFB risk.

Variable category	Variable	Explanation of odds ratio	Proposed significance for beekeepers
Demographic	Beekeeper Size	The odds of being in a higher AFB risk category are greater for large-scale beekeepers than for small-scale beekeepers	Potential selection bias during beekeeper enrolment Large size of operations may increase likelihood of close proximity to sources of spores
General Management	Indoor Overwintering of Hives	The odds of being in a higher AFB risk category are greater for beekeepers overwintering a large proportion of hives indoors than beekeepers overwintering fewer hives indoors	Biosecuirty must be considered with indoor overwintering Return colonies to apiaries they are collected from; identify hives for traceability Reduce any tendency toward the indoor overwintering of weak colonies
	Purchase of Used Equipment	The odds of being in a higher AFB risk category are greater for beekeepers that have purchased used equipment than for those beekeepers who have not	Minimize the purchase of used equipment whenever possible If used equipment must be purchased, ensure professional inspection and/or testing Ensure appropriate quarantine and identification to improve traceability
Prevention of AFB	Current Antibiotic Use	The odds of being in a higher AFB risk category are greater for those beekeepers that are currently using or have recently used antimicrobials than for those that have never used antimicrobials or have not used them recently	Current or recent use likely reflective of recent experience with or known risk of AFB
	Confidence in AFB Recognition	The odds of being in a higher AFB risk category are greater for beekeepers that are very confident at recognizing AFB than those that are somewhat or not confident at recognizing AFB	Ability to recognize AFB likely reflective of previous experience with AFB
	Movement of Colonies between Apiaries	The odds of being in a higher AFB risk category are greater for beekeepers that move honey-producing colonies between apiaries than those who do not	Minimize the movement of honey-producing colonies between apiaries whenever possible If colonies must be moved, ensure appropriate identification to improve traceability

Our final model identified the indoor overwintering of bees as a general management variable predictive of moderate to high risk of future AFB disease. In western Canada and other regions of similar climate, some beekeepers elect to move colonies indoors during the cold months of winter^44^. In our univariable analysis, the odds of being in a higher risk category increased for every 10% increase in the proportion of hives overwintered indoors. To the best of our knowledge, this is the first report of a link between indoor overwintering and future AFB risk. Importantly, we do not believe the act of indoor overwintering itself to be a direct cause of AFB. Rather, we suspect this finding may reflect a tendency to bring relatively weaker colonies indoors that would otherwise perish if left outdoors overwinter. These weaker colonies may, in turn, be predisposed to AFB if considerable numbers of spores are present within the hive and could subsequently act as a source of infection. This risk could be avoided if weak colonies were left outdoors or quarantined elsewhere. Alternatively, the overwintering of colonies indoors may further reflect issues with biosecurity, as beekeepers do not necessarily return individual hives to the same apiary that they were collected from in the fall. We hypothesize that improving biosecurity in indoor overwintering protocols, including identifiers that ensure consistent return of hives to their apiary of origin, may help to mitigate this source of risk.

We found that there were increased odds of a beekeeper being identified as large-scale relative to small-scale in higher AFB risk categories. This may partially be the result of selection bias. Candidate L beekeepers were identified primarily through publicly available listings of mandatory registration information for their operations. Conversely, S beekeeper candidates were identified largely through solicitation in municipal beekeeping clubs, which reflects those S beekeepers with a high level of interest and motivation in apiculture and honey bee health. Consequently, we were unable to sample the demographic of S beekeepers uninterested in attending such meetings. Alternatively, the association of L beekeepers with higher risk categories of AFB may relate to the sheer size of some of these beekeeping operations, each of which manage large numbers of apiaries spread over a wide geographic area. De Graaf et al. (2001) demonstrated that honey samples from apiaries in close proximity to apiaries with recent AFB were at a higher risk of being contaminated by spores^30^. This is probably the result of robbing behavior by nearby bees^46^. We speculate that the sprawling distribution of some of these operations may increase their likelihood of being in close proximity of other beekeepers with recent AFB or, at the very least, high spore contamination. Indeed, we identified one L beekeeper whose spore concentrations increased from low risk to moderate risk between 2019 and 2020, an increase that was attributed to the presence of a confirmed case of AFB within a nearby beekeeper’s hive.

The remaining variables represented in our final model (use of current antimicrobial metaphylaxis, confidence in AFB recognition, and historical occurrence of AFB), although essential to explain variation in our dataset, may be reflective of the consequences of AFB rather than true predictive risk factors (Table 3). We suspect that any beekeeper with a recent history of AFB would consequently be more likely to recognize its clinical signs as opposed to a beekeeper who has never had experience with the disease. Similarly, such beekeepers would also be more likely to be using antimicrobials in response to recognition of a problem with AFB.

Overall, our final predictive model was capable of explaining 25.8% of variation, as approximated by pseudo R-squared. Although the ability of the final model to discriminate between low risk and higher risk (i.e., moderate to high risk) categories was excellent, the model was relatively poor at predicting beekeepers in moderate and high risk categories and could not differentiate between moderate and high risk based on our threshold of 100 spores per gram of honey. Nevertheless, we advocate the inclusion of this moderate to high risk threshold, as it stands to reason that a beekeeper with a maximum spore concentration of two spores per gram of honey would require less intensive intervention than one with hundreds to thousands of spores per gram of honey. Taken together, the low pseudo R-squared value of the model and poor differentiation of higher risk categories is likely reflective of the absence of important variables that could further explain variation in our dataset. One such variable may be apiary proximity to areas of recent or active AFB, as multiple other studies have identified this as an important risk factor^30,43,46,47^. We asked beekeepers to enumerate nearby beekeepers to determine beekeeper density as a proxy of nearby AFB, however, poor response rates in our questionnaire precluded evaluation. Additionally, de Graaf et al. (2001) identified brood comb replacement as an important risk factor for the contamination of honey by *P. larvae* spores^30^. We assessed comb replacement and found no statistical significance (N = 85, p = 0.97); however, we measured comb replacement as a continuous variable (i.e., 0 to 100%), whereas de Graaf et al. (2001) assessed comb replacement as a binary ‘yes’ or ‘no’. The near uniform incorporation of some amount of brood comb replacement amongst participant beekeepers in our study, especially amongst L beekeepers, precludes evaluation of this variable in a comparable way to de Graaf et al. (2001).

In conclusion, we have developed a logistically feasible means for Saskatchewan beekeepers of all demographics, and particularly those that manage large-scale commercial beekeeping operations, to assess their risk of future AFB disease at the apiary or operation level. We suspect that these findings are also relevant for beekeepers in other western Canadian provinces with similar beekeeping management practices. Since the great majority of commercial beekeeping operations in this study were stationary, it is not clear if our thresholds are applicable to commercial beekeeping operations in the USA, as approximately 75% of their commercial honey bee colonies are subject to migratory management. In addition, through predictive modelling, we have identified management practices that potentially increase the risk of AFB in antibiotic-reliant apiculture, thereby providing beekeepers and their health management teams (i.e., provincial specialists in apiculture and veterinarians) targets for intervention to mitigate risk. Together, an understanding of (i) AFB risk through spore concentrations in pooled, extracted honey, and (ii) management practices that contribute to risk in antibiotic-reliant management systems, will improve evidence-based decision making in western Canadian apiculture in relation to the prevention and management of AFB. In turn, this will promote a shift toward more judicious use of antimicrobials, helping to ensure the future sustainability of this sector by reducing antimicrobial resistance.

### Recommendations for beekeepers and their health management teams in western Canada

1. Identify one or more apiaries (honey bee yards) for sampling during the final honey pull of the season. Sampling late into the honey-producing season is recommended to ensure sampling is as far removed from the last application of antibiotics as possible. If sampling more than one apiary, ensure selected apiaries are geographically separate from one another and representative of the entire beekeeping operation.
2. During extraction of honey from frames, collect a total of three honey samples from each selected apiary. Each honey sample must come from a separate spin cycle/extractor load to ensure that the same frames are not repeatedly tested. Each sample should fill a 50 g container.
3. Submit samples to the appropriate diagnostic laboratory for detection and quantification of *P. larvae* spores and assignment of risk category for future AFB disease.
4. If a beekeeper tests as moderate to high risk for future AFB disease, they should consult and collaborate with their health management team (i.e., provincial specialist in apiculture and veterinarian) to perform thorough inspections of affected apiaries, apply antimicrobials as needed, and identify management practices requiring intervention (see Table 1). Special attention should be paid to the following:
  Biosecurity measures associated with the labeling and movement of hives during indoor overwintering
  Selection criteria used for indoor overwintering candidates (for example, avoiding overwintering weak colonies)
  Biosecurity measures associated with the labeling and movement of honey-producing colonies between apiaries
  Biosecurity measures associated with the purchase and integration of used equipment from other beekeepers
5. If a beekeeper tests as low risk for future AFB disease and does not practice any high-risk activities (see list in point 4), they may temporarily discontinue antimicrobial metaphylaxis while maintaining other components of their integrated pest management strategies (ex. frequent, thorough visual inspection of brood chambers).
6. Testing of pooled, extracted honey should continue on an annual basis in addition to other integrated pest management strategies to allow for ongoing, evidence-based decision-making between the beekeeper and their health management team. Adjustments to preventative management practices may be made as indicated by test results.

## Supplementary Information

Supplementary Information 1.

Supplementary Information 2.

Supplementary Information 3.

## Data Availability

All data generated or analysed during this study are included in this published article (and its supplementary information files).
